# The combination of hand grip strength and modified Glasgow prognostic score predicts clinical outcomes in patients with liver cancer

**DOI:** 10.3389/fnut.2023.1062117

**Published:** 2023-02-27

**Authors:** Yue Chen, Guo-Tian Ruan, Jin-Yu Shi, Tong Liu, Chen-An Liu, Hai-Lun Xie, Meng-Meng Song, Zi-Wen Wang, Chun-Lei Hu, He-Yang Zhang, Xiao-Wei Zhang, Hai-Ying Tian, Yi-Zhong Ge, Ming Yang, Yu-Ying Liu, Shi-Qi Lin, Xiao-Yue Liu, Xin Zheng, Kun-Hua Wang, Ming-Hua Cong, Xian Shen, Xin Wang, Li Deng, Han-Ping Shi

**Affiliations:** ^1^Department of Gastrointestinal Surgery, Department of Clinical Nutrition, Beijing Shijitan Hospital, Capital Medical University, Beijing, China; ^2^Key Laboratory of Cancer FSMP for State Market Regulation, Beijing, China; ^3^Clinical Medical College, Yunnan University, Kunming, China; ^4^General Surgery Clinical Medical Center of Yunnan Province, Kunming, China; ^5^Comprehensive Oncology Department, National Cancer Center, Cancer Hospital, Chinese Academy of Medical Sciences and Peking Union Medical College, Beijing, China; ^6^The Second Affiliated Hospital of Wenzhou Medical University, Wenzhou, Zhejiang, China

**Keywords:** hand grip strength, inflammation, mGPS, liver cancer, nomogram

## Abstract

**Purpose:**

Previous studies have shown that both hand grip strength (HGS) and the modified Glasgow Prognostic Score (mGPS) are associated with poor clinical outcomes in patients with liver cancer. In spite of this, no relevant studies have been conducted to determine whether the combination of HGS and mGPS can predict the prognosis of patients with liver cancer. Accordingly, this study sought to explore this possibility.

**Methods:**

This was a multicenter study of patients with liver cancer. Based on the optimal HGS cutoff value for each sex, we determined the HGS cutoff values. The patients were divided into high and low HGS groups based on their HGS scores. An mGPS of 0 was defined as low mGPS, whereas scores higher than 0 were defined as high mGPS. The patients were combined into HGS-mGPS groups for the prediction of survival. Survival analysis was performed using Kaplan–Meier curves. A Cox regression model was designed and adjusted for confounders. To evaluate the nomogram model, receiver operating characteristic curves and calibration curves were used.

**Results:**

A total of 504 patients were enrolled in this study. Of these, 386 (76.6%) were men (mean [SD] age, 56.63 [12.06] years). Multivariate analysis revealed that patients with low HGS and high mGPS had a higher risk of death than those with neither low HGS nor high mGPS (hazard ratio [HR],1.50; 95% confidence interval [CI],1.14–1.98; *p* = 0.001 and HR, 1.55; 95% CI, 1.14–2.12, *p* = 0.001 respectively). Patients with both low HGS and high mGPS had 2.35-fold increased risk of death (HR, 2.35; 95% CI, 1.52–3.63; *p* < 0.001). The area under the curve of HGS-mGPS was 0.623. The calibration curve demonstrated the validity of the HGS-mGPS nomogram model for predicting the survival of patients with liver cancer.

**Conclusion:**

A combination of low HGS and high mGPS is associated with poor prognosis in patients with liver cancer. The combination of HGS and mGPS can predict the prognosis of liver cancer more accurately than HGS or mGPS alone. The nomogram model developed in this study can effectively predict the survival outcomes of liver cancer.

## Introduction

Globally, primary liver cancer is the fifth most common cancer and the third leading cause of cancer death (1). In 2018, the global mortality rate for liver cancer reached about 8.5 per 100,000 individuals (2). There is a high incidence of liver cancer in East Asia (3).

Patients with liver cancer are often malnourished because the liver is involved in nutrient metabolism. The poor prognosis of liver cancer has also been linked to impaired nutritional status in some studies (4). The poor nutritional status associated with liver cancer affects a patient’s quality of life, the intensity of treatment, and the outcome of disease. Therefore, it is critical to identify nutritional indicators that are easily measured in clinical settings for predicting the prognosis of liver cancer.

Muscle mass is a common nutritional indicator and is often expressed in terms of skeleton muscle mass (SMM). However, SMM is assessed using computed tomography, which is expensive and exerts a significant financial burden on the patient (4). Hand grip strength (HGS) is another indicator that is often used to evaluate muscle mass and is easily measured in clinical settings. Complication-free survival is significantly worse in patients with low HGS, according to previous studies (5). A recent study suggested that HGS is a biomarker for aging and predisposition to various diseases that lead to death (6). A study based on the UK Biobank showed that HGS is associated with mortality from cancer and other disease (7). Victoria et al. found that HGS is a useful tool for diagnosing malnutrition and has a predictive value for six-month mortality in inpatients with cancer (8). Therefore, we speculated that HGS could reflect muscle mass and play a role in predicting the prognosis of liver cancer.

Using a combination of C-reactive protein and albumin levels, the modified Glasgow Prognostic Score (mGPS) provides a prognostic score for patients who have cancer. The mGPS reflects the nutritional and inflammatory status of a patient. It has been shown to have prognostic value for lung, gastrointestinal, and renal cancers, independent of the tumor stage (9). In addition, the mGPS has been found to be significantly associated with sarcopenia in patients with gastric and esophageal cancers (10). Some studies have shown that the systemic inflammatory response evidenced by the mGPS is common in large patient cohorts. Compared with other biochemical parameters, mGPS is a strong prognostic factor, independent of tumor site (9). It has also been suggested that the mGPS may be an independent prognostic factor for liver cancer (11).

Several studies on the prognostic values of HGS and mGPS have been conducted. To our knowledge, there have been no studies to investigate whether liver cancer outcomes can be predicted by combining HGS and mGPS. Therefore, the aim of this study was to investigate the roles of HGS, mGPS, and HGS-mGPS in predicting the prognosis of liver cancer.

## Methods

### Study population and design

A retrospective analysis of liver cancer patient data from multiple Chinese clinical centers from April 2013 to September 2019 was conducted in this study. The inclusion criteria for this study were as follows: age ≥ 18 years old; the primary tumor was diagnosed as hepatocellular carcinoma; duration of hospitalization ≥2 days; and provision of a signed consent form. A minimum of 18 years old, a hospital stay of 2 days, refusal to sign the consent form, and admission to the intensive care unit at the beginning of the recruitment process were the exclusion criteria. In accordance with the principles of the Declaration of Helsinki, the study was approved by the medical ethical review committee of the hospital where it was conducted.

### Patient characteristics

The following information were extracted from the patient records: sex; age; tumor stage; surgery, chemotherapy, and radiotherapy data; HGS; trigeminal skinfold thickness (TSF); body mass index (BMI); and total protein, albumin, C-reactive protein (CRP), aspartate aminotransferase, and alanine transaminase levels.

### Laboratory and anthropometric measurements

All blood tests were conducted within 48 h of hospitalization after patients had fasted for at least 9 hours.

Hand grip strength was measured using an electronic hand-held dynamometer (CAMRY, EH101 model, Guangdong, China). Patients were instructed to stand comfortably and perform three maximal isometric contractions with the nondominant hand 30 s apart. BMI was calculated as follows: BMI (kg/m^2^) = weight (kg)/height^2^ (m^2^). TSF involves creating a skinfold by grasping the patient’s skin 2 cm above the midpoint of the right upper arm with the thumb and index finger. Calipers were then placed at the midpoint of the skinfold for the measurement of TSF (12).

### Definition of variables and evaluation of outcomes

We used the log-rank method to determine the HGS cutoff values for men and women separately. The optimal HGS cutoff values for men and women were 28.3 and 18.6, respectively (Supplementary Figure S2). The patients were classified into low or high HGS groups based on these cutoff values.

An mGPS of 0 is determined as a CRP level < 10 mg/L and an albumin level > 35 g/L, a score of 1 as a CRP level > 10 mg/L or an albumin level < 35 g/L, and a score of 2 as a CRP level > 10 mg/L and an albumin level < 35 g/L (13). An mGPS of 0 was defined as a low mGPS, whereas scores higher than 0 were defined as a high mGPS.

Our method for gathering follow-up records was to obtain them strictly according to the established content from all telephone consultations and follow-ups in outpatient clinics. Observed outcomes were overall survival (OS), which is the time between the first diagnosis of cancer and death, withdrawal from study, or last follow-up.

### Statistical analysis

When a variable has a normal distribution, the mean + standard deviation is calculated, while variables with a non-normal distribution are calculated by median (interquartile range). The categorical baseline characteristics of the patients were compared using the chi-squared test and are expressed as numbers (percentages). The independent Student’s *t*-test or rank test and the *χ*^2^ test were used for the comparison of continuous variables and categorical data, respectively, between the two groups.

For the survival analyses, we cross-classified the low or high HGS and low or high mGPS groups into four categories (only low HGS, only high mGPS, both, and neither [reference]). A Kaplan–Meier survival curve was calculated using the Kaplan–Meier method. To assess the risk of mortality and value reliability, we calculated hazard ratios (HRs) and 95% confidence intervals (CIs). In the multivariate Cox regression model for the risk of mortality, model 0 was an unadjusted model, model 1 was adjusted for age, sex, and tumor stage, and model 2 was adjusted for age, sex, tumor stage, surgery, radiotherapy, chemotherapy, TSF, smoking, and alcohol consumption. The nomogram was used to establish a prediction model based on HGS-mGPS and survival probability. Nomogram accuracy was evaluated using the area under the curve (AUC) and calibration curves. All statistical analyses were performed by the R Studio statistical software (version 4.2.0). Two-sided *p*-values <0.05 were considered statistically significant.

## Results

### Patients characteristics

A total of 798 patients with liver cancer were identified in INSCOC database. After excluding patients with missing data, 504 patients were included in this study (Supplementary Figure S1). The study population comprised 386 men (76.6%) and 118 women (23.4%), and their mean age was 56.63 ± 12.06 years (Table 1). Supplementary Table S1 summarizes the baseline characteristics of the patients as determined by their HGS and mGPS categories. The results showed that low HGS was significantly associated with increased CRP level (*p* < 0.001). In addition, the results indicated that mGPS was associated with HGS (*p* < 0.001). HGS and mGPS were both associated with tumor stage (*p* = 0.001 and *p* < 0.001, respectively).

**Table 1:** Baseline characteristics of patients with liver cancer (*N* = 504).

	Patients (504)
Sex, *n* (%)	
Men	386 (76.6)
Women	118 (23.4)
Age (year)	56.63 ± 12.06
Tumor stage, *n* (%)	
I	77 (15.3)
II	93 (18.5)
III	102 (20.2)
IV	232 (45.9)
Surgery, *n* (%)	
Yes	204 (40.5)
No	300 (59.5)
Chemotherapy, *n* (%)	
Yes	86 (17.1)
No	419 (83.0)
Radiotherapy, *n* (%)	
Yes	18 (3.6)
No	487 (96.4)
Liver cirrhosis, *n* (%)	
Yes	107 (21.2%)
No	397 (78.8%)
Smoking *n* (%)	
Yes	122 (24.2)
No	106 (21.0)
Other	276 (54.8)
Alcohol *n* (%)	
Yes	130 (25.8)
No	374 (74.2)
BMI (kg/m^2^)	22.37 ± 3.23
Total protein (g/L)	68.36 ± 8.09
Albumin (g/L)	37.02 ± 6.14
TSF (mm)	14.00 [10.00, 20.00]
HGS (kg)	26.30 [20.60, 32.00]
CRP (mg/L)	16.82 [4.40, 34.70]
AST (U/L)	40.00 [27.00, 75.40]
ALT (U/L)	35.00 [23.10, 58.80]

Continuous variables are presented as mean ± standard deviation (SD). Meanwhile, TSF, HGS, CRP, AST and ALT are presented as the median (interquartile range). Categorical variables are presented as numbers and percentages. Differences in normally and non-normally distributed baseline characteristics were compared using the chi-square test or *t*-test and Wilcoxon rank sum test, respectively. TSF, triceps skinfold thickness; HGS, handgrip strength; BMI, body mass index; CRP, C reactive protein; AST, aspartate aminotransferase; ALT, Alanine aminotransferase.

### Relationship between hand grip strength, modified Glasgow prognostic score, hand grip strength-Glasgow prognostic score, and overall survival

Based on Cox regression models adjusted for potential confounders, Table 2 shows the association between each indicator and OS in patients with liver cancer. Death risk was 50% higher in the low HGS group than in the high HGS group (adjusted HR = 1.50; 95% CI: 1.14–1.98; adjusted *p* = 0.004). Continuous HGS, however, did not significantly affect OS (adjusted HR = 0.99; 95% CI: 0.97–1.01; adjusted *p* = 0.197). There was a higher risk of death in the high mGPS group compared to the low mGPS group in regards to mGPS (adjusted HR = 1.55; 95% CI: 1.14–2.12; adjusted *p* = 0.001). HGS-mGPS showed that patients with both low HGS and high mGPS had a significantly higher mortality risk than those with neither (adjusted HR = 2.35; 95% CI: 1.52–3.63; adjusted *p* < 0.001).

**Table 2:** Association of each indicator and overall survival in patients with liver cancer according to cox regression models adjusted for potential confounders.

	*N*	Model 0		Model 1		Model 2	
		HR (95%CI)	*p*-value	HR (95%CI)	*p*-value	HR (95%CI)	*p*-value
HGS							
As continues	504	**0.98 (0.96–0.99)**	**<0.001**	0.99 (0.97–1.00)	0.073	0.99 (0.97–1.01)	0.197
High HGS	256	Ref.		Ref.		Ref.	
Low HGS	248	**1.90 (1.48–2.44)**	**<0.001**	**1.57 (1.20–2.04)**	**0.001**	**1.50 (1.14–1.98)**	**0.004**
mGPS							
0	148	Ref.		Ref.		Ref.	
1	202	**1.88 (1.36–2.61)**	**<0.001**	**1.42 (1.02–1.98)**	**0.040**	1.40 (1.00–1.97)	0.055
2	154	**2.48 (1.77–3.47)**	**<0.001**	**1.93 (1.37–2.70)**	**<0.001**	**1.84 (1.30–2.61)**	**0.001**
Low mGPS (mGPS = 0)	148	Ref.		Ref.		Ref.	
High mGPS (mGPS = 1 or 2)	356	**2.12 (1.57–2.86)**	**<0.001**	**1.58 (1.16–2.14)**	**0.004**	**1.55 (1.14–2.12)**	**0.001**
HGS-mGPS							
Low risk (Neither)	95	Ref.		Ref.		Ref.	
Median risk 1 (Only low HGS)	53	**2.13 (1.25–3.64)**	**0.006**	**2.07 (1.21–3.56)**	**0.008**	**2.01 (1.16–3.47)**	**0.013**
Median risk 2 (Only high mGPS)	161	**2.19 (1.42–3.37)**	**<0.001**	**1.86 (1.20–2.86)**	**0.005**	**1.84 (1.19–2.85)**	**0.006**
High risk (Both)	195	**3.60 (2.38–5.44)**	**<0.001**	**2.49 (1.63–3.81)**	**<0.001**	**2.35 (1.52–3.63)**	**<0.001**

Data are presented as hazard ratios (95% confidence intervals). Model 0: unadjusted. Model 1: Adjusted for age, sex, tumor stage. Model 2: Adjusted for age, sex tumor stage, surgery, radiotherapy, chemotherapy, TSF, smoking and alcohol. HGS, hand grip strength; mGPS, modified Glasgow Prognostic Score; HGS-mGPS, the combination of hand grip strength and modified Glasgow Prognostic Score. Bold values indicate statistically significant level (*p* < 0.05).

**Figure 1:**
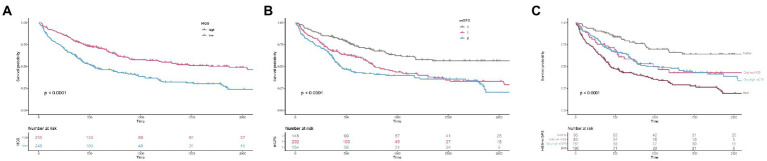
The Kaplan–Meier survival curves of HGS, mGPS, and HGS-mGPS. **(A)** HGS; **(B)** mGPS; **(C)** HGS-mGPS. HGS, hand grip strength; mGPS, modified Glasgow Prognosis Score.

Figure 1 shows the Kaplan–Meier curves for patients in different HGS, mGPS, and risk groups. The OS of patients with high HGS was significantly better than that of patients with low HGS (*p* < 0.001). Patients with a high mGPS had worse OS than those with a low mGPS (*p* < 0.001). Patients in the high-risk group (both low HGS and high mGPS) had the worst OS, whereas those in the low-risk group (neither low HGS nor high mGPS) had the best OS (*p* < 0.001).

### Stratified analysis

Stratified analyses were conducted to evaluate the relationship between HGS-mGPS and the HR of OS in various subgroups (Table 3). Among liver cancer patients, the association between OS and HGS-mGPS was not modified by age, stage, alcohol intake, or BMI (Table 3). Among patients with both low HGS and high mGPS, even younger patients (<65 years; adjusted HR, 2.16; 95% CI, 1.30–3.60, adjusted *p* = 0.003) with no history of no alcohol consumption (adjusted HR, 2.44; 95% CI, 1.45–4.13, adjusted *p* = 0.001) had more than a two-fold increased risk of death compared with patients with similar characteristics but with neither low HGS nor high mGPS. Subgroup analyses revealed no previous interaction between these factors and HGS-mGPS.

**Table 3:** HGS, mGPS, and liver cancer overall survival stratified by body mass index, tumor stage, age, sex, smoking, and alcohol consumption.

Stratification variable	Univariate analysis		Multivariate analysis		*p* for interaction
	HR (95%CI)	*p*-value	HR (95%CI)	*p*-value	
Tumor stage					0.478
I	3.81 (0.92–15.74)	0.065	4.01 (0.79–20.37)	0.094	
II	2.96 (1.18–7.43)	0.021	3.26 (1.10–9.66)	0.033	
III	2.70 (1.03–7.06)	0.043	2.94 (1.11–7.77)	0.030	
IV	2.66 (1.45–4.90)	0.002	2.26 (1.19–4.29)	0.013	
Age					0.936
<65	3.17 (1.95–5.13)	<0.001	2.16 (1.30–3.60)	0.003	
≥65	3.98 (1.57–10.09)	0.004	3.48 (1.31–9.19)	0.012	
Sex					0.207
Man	3.23 (2.04–5.11)	<0.001	1.99 (1.22–3.25)	0.006	
Woman	5.47 (2.08–14.39)	0.001	4.04 (1.46–11.17)	0.007	
Smoking					0.637
Yes	2.26 (1.10–4.66)	0.027	2.00 (0.89–4.52)	0.095	
No	3.50 (0.83–14.78)	0.088	2.90 (0.61–13.68)	0.179	
Other	4.72 (2.69–8.28)	<0.001	2.59 (1.43–4.72)	0.002	
Alcohol consumption					0.327
Yes	2.36 (1.09–5.12)	0.029	2.01 (0.91–4.46)	0.084	
No	4.12 (2.52–6.74)	<0.001	2.44 (1.45–4.13)	0.001	
BMI					1.000
<18.5	1.45 (0.43–4.85)	0.550	1.39 (0.31–6.32)	0.669	
18.5–24.9	3.46 (2.12–5.65)	<0.001	2.28 (1.36–3.82)	0.002	
≥25	1.77 (1.28–2.44)	0.001	1.55 (1.08–2.22)	0.017	

Cox proportional hazards multiple models adjust for BMI categories (<18.5,18.5–24.9,≥25), at-diagnosis values of age (years), sex (man or woman), tumor stage (I, II, III or IV), surgery (yes or no), chemotherapy (yes or no), radiotherapy, smoking (yes or no), alcohol (yes or no) and TSF, unless stratified by those variables. All HRs compare patients with low HGS and high mGPS vs patients with high HGS and low mGPS.

### Nomogram and evaluation

**Figure 2:**
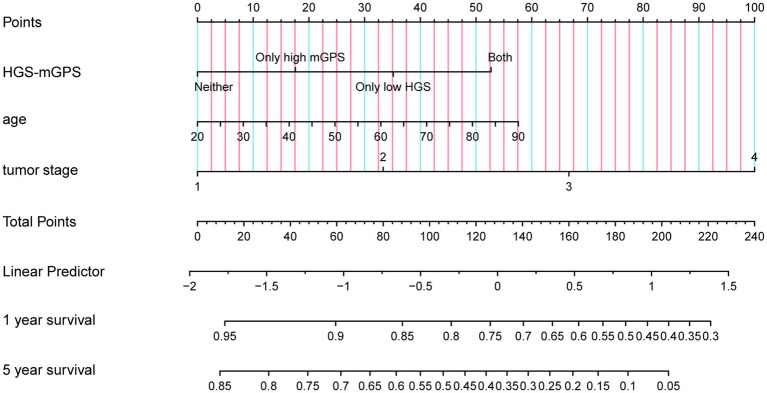
A proposed Nomogram for predicting 1 year and 5 year survival probability of patients with liver cancer. HGS-mGPS, the combination of HGS and mGPS.

COX regression analysis was used to investigate the prognostic factors of liver cancer patients. The result showed that age and tumor stage were independent risk factors for them (Supplementary Table S2). The nomogram was constructed using independent prognostic factors (age, tumor stage, and HGS-mGPS; Figure 2). The AUC for HGS-mGPS was 0.623 (Supplementary Figure S3). This result indicated that HGS-mGPS has predictive value for liver cancer. Based on the nomogram, the probability of survival in liver cancer patients is predicted in Figure 2. The total score was determined based on the individual scores calculated using the nomogram. The total risk points of most patients in the present study ranged from 0 to 240. The calibration curves reflected good agreement between 1-year and 5-year OS and the predictions from the nomogram (Supplementary Figure S4).

### Sensitivity analysis

Given that HGS-mGPS has prognostic value for liver cancer, a sensitivity analysis was performed (Table 4). Table 4 shows the results of the sensitivity analysis after patients who died less than 6 months from the start of the first investigation were excluded. High-risk groups of HGS-mGPS had a significantly greater risk of death than low-risk groups (adjusted HR = 2.34; 95% CI: 1.42–3.87; adjusted *p* = 0.001).

**Table 4:** Sensitive analysis (Excluded patients who died in 180 days) (*n* = 413).

	*N*	Model 0		Model 1		Model 2	
		HR (95%CI)	*p*-value	HR (95%CI)	*p*-value	HR (95%CI)	*p*-value
HGS							
As continues	413	0.98 (0.97–1.00)	0.051	0.99 (0.97–1.01)	0.236	0.99 (0.97–1.01)	0.192
High HGS	227	Ref.		Ref.		Ref.	
Low HGS	186	**1.71 (1.26–2.31)**	**<0.001**	**1.44 (1.05–1.98)**	**0.023**	**1.55 (1.11–2.17)**	**0.010**
mGPS							
0	113	Ref.		Ref.		Ref.	
1	163	**1.94 (1.31–2.86)**	**0.001**	**1.47 (0.99–2.19)**	**0.056**	1.36 (0.91–2.04)	0.132
2	117	**2.42 (1.61–3.64)**	**<0.001**	**1.93 (1.28–2.90)**	**0.002**	**1.99 (1.31–3.02)**	**0.001**
Low mGPS (mGPS = 0)	133	Ref.		Ref.		Ref.	
High mGPS (mGPS = 1 or 2)	280	**2.13 (1.49–3.04)**	**<0.001**	**1.65 (1.14–2.37)**	**0.007**	**1.86 (1.29–2.68)**	**0.001**
HGS-mGPS							
Low risk (Neither)	89	Ref.		Ref.		Ref.	
Median risk 1 (Only low HGS)	44	**1.91 (1.01–3.61)**	**0.046**	1.86 (0.98–3.54)	0.057	**2.02 (1.05–3.87)**	**0.035**
Median risk 2 (Only high mGPS)	138	**2.23 (1.36–3.63)**	**0.001**	**1.87 (1.14–3.06)**	**0.013**	**1.79 (1.09–2.94)**	**0.022**
High risk (Both)	142	**3.26 (2.02–5.27)**	**<0.001**	**2.30 (1.40–3.87)**	**0.001**	**2.34 (1.42–3.87)**	**0.001**

Data are presented as hazard ratios (95% confidence intervals). Model 0: unadjusted. Model 1: Adjusted for age, gender, tumor stage. Model 2: Adjusted for age, sex tumor stage, surgery, radiotherapy, chemotherapy, TSF, smoking and alcohol. HGS, handgrip strength; mGPS, modified Glasgow Prognostic Score; HGS-mGPS, combination of HGS and mGPS; HR, hazard ratio; CI, confidence interval; *p*, probability. Bold values indicate statistically significant level (*p* < 0.05).

## Discussion

In this multicenter study, we investigated the prognosis values of HGS, mGPS, and HGS-mGPS for liver cancer. According to the results, HGS and mGPS are independently associated with liver cancer prognoses, but their combination negatively correlates with OS. In addition, the nomogram model we developed that incorporated HGS-mGPS and other factors. The model effectively predicted the survival outcomes of liver cancer.

A previous study showed that absolute HGS is associated with liver cancer (14). This study showed, however, that continuous HGS values in liver cancer patients are not significantly associated with all-cause mortality, especially after taking into account confounding variables. We calculated the HGS cutoff values for men and women separately to define low HGS. Low HGS was associated with poor survival probability compared to high HGS based on Cox regression analysis and Kaplan–Meier curves. HGS is strongly correlated with leg muscle strength and is a valid marker of overall limb muscle strength across all age groups. It has also been suggested that HGS is a marker of nutritional status (15, 16). According to a review published in the Lancet, muscle function is important for the diagnosis of sarcopenia, and HGS is one of the commonly used measures (17). However, HGS can only reflect muscle condition and still has limitations. HGS is easily affected by other factors when predicting prognosis. The 2016 Global Leadership Initiative on Malnutrition criteria states that at least one phenotypic criterion and one etiological criterion are required in the diagnosis of malnutrition (18). Therefore, we included inflammatory factors in the present study.

The three-scale mGPS is based on a combination of C-reactive protein and albumin levels and is graded from 0 to 2. The mGPS has been validated worldwide, and has been proven to have an independent prognostic value for various types and stages of cancer (19). The mGPS has also proven useful as a prognostic tool at the time of diagnosis, as well as in patients with a possible ongoing malignancy. The present study showed that mGPS was significantly linked to liver cancer mortality overall. Patients with an mGPS of 3 had a higher risk of death than those with an mGPS of 1. However, in the survival analysis, the risk of death in patients with an mGPS of 2 was not significantly different from that in patients with an mGPS of 3. Therefore, we defined patients with mGPS of 2 or 3 as those with high mGPS.

We combined the HGS and mGPS groups to form a new index, HGS-mGPS. The HGS-mGPS categories included the low-risk, median-risk, and high-risk groups. The risk of death was significantly different among the four groups. As a result of Cox regression and survival analyses, it was found that patients in the high-risk group were at the greatest risk of death. The combination of HGS and mGPS can predict the prognosis of liver cancer more accurately than HGS or mGPS alone. We speculate that the underlying mechanism for this finding is the synergistic effect of muscle loss and a high inflammatory load. A review suggested that the prevalence of sarcopenia is approximately 39% in patients with hepatocellular carcinoma. The mechanism is complex, but it is well established that chronic liver disease can trigger muscle atrophy and structural changes in skeletal muscle, and skeletal muscle compartments contribute to the progression of liver disease (20). In addition, muscle loss leads to insulin resistance and increases the activity of IGF-1, which can regulate hepatocyte proliferation (21). No matter what stage of cancer a patient is at, low muscle mass negatively impacts physical function, quality of life, surgical complications, cancer progression, and reduced possibility of survival (22). Reduced muscle mass increases the inflammatory burden, which further promotes muscle loss. Inflammatory markers in the blood have been shown to exacerbate the loss of muscle strength in previous studies (23). Our study found that lower HGS was significantly associated with higher CRP levels. This result is consistent with previous study (24). CRP changes the expression of proto-oncogenes and suppressor genes and immune regulation through different pathways, affecting cancer cell proliferation, migration, invasion, chemotherapy resistance and immune system resistance (25). Researchers have previously shown a link between higher CRP levels and lower walking speeds and grip strength (26). CRP and albumin may be active mediators of both hepatocellular cancer development and a more aggressive phenotype, rather than merely passive reflections of inflammatory processes. In addition, CRP level seems to reflect mechanism of hepatocellular cancer development (27). Muscle and inflammation are both related to the prognosis of liver cancer, and they interact with each other. Therefore, the combination of HGS and mGPS can predict the outcome of liver cancer more accurately than HGS and mGPS alone and is not susceptible to other confounding factors. In addition, the acquisition of HGS, CRP, and albumin data is simpler and more economical than other expensive examinations.

Malnutrition is a significant risk for patients with liver cancer since the liver is the organ responsible for nutrient metabolism. Malnutrition is common in patients with liver cirrhosis and is associated with mortality and a reduced quality of life (28). Poor nutrition further exacerbates muscle loss which in turn activates the inflammation cascade. Therefore, it is important to pay sufficient attention to malnutrition in patients with liver cancer and note that nutritional status is closely related to their clinical performance. A meta-analysis showed that nutritional intervention can significantly improve the nutritional statuses of patients with gastric cancer (29). However, only 33.9% of physicians follow the recommendations in the oncology section of the ESPEN guidelines (30). A recent review showed that in patients at risk of developing liver cancer, the chance of progression to cachexia is as high as 50% (31). Therefore, more attention should be paid to the nutritional statuses of patients with liver cancer who show low HGS or increased blood inflammatory markers. In addition, it should be noted that improving muscle mass and systemic inflammation can improve the prognosis of patients with liver cancer.

This study has some limitations. First, the study only included Asians, in addition to having a small sample size. It is therefore necessary to conduct future studies with patient samples from diverse ethnicities in order to confirm the findings of the present study. Second, no additional treatment modalities were analyzed in this study. Thirdly, there was a lack of specific assessment of cirrhosis. Finally, the nomogram developed in this study requires further external validation.

Despite these limitations, this study demonstrated the clinical value of HGS-mGPS and its correlation with the outcomes of liver cancer. To our knowledge, this is the first multicenter study in which the value of the combination of HGS and mGPS for predicting the survival of patients with liver cancer was explored.

## Conclusion

This study showed that low HGS and high mGPS are associated with poor prognosis in patients with liver cancer. Furthermore, patients with both low HGS and high mGPS have a higher mortality risk than those with neither low HGS nor high mGPS. The nomogram model developed in the present study can effectively predict the outcomes of liver cancer.

## Data availability statement

The raw data supporting the conclusions of this article will be made available by the authors, without undue reservation.

## Ethics statement

This study followed the Helsinki declaration. All participants signed an informed consent form, and this study was approved by the Institutional Review Board of each hospital (Registration number: ChiCTR1800020329).

## Author contributions

YC wrote the manuscript. YC, G-TR, and J-YS analyzed and interpreted the patient data. YC, G-TR, J-YS, C-AL, TL, and H-PS made substantial contributions to the conception, design, and intellectual content of the studies. All authors read and approved the final manuscript.

## Funding

This work was supported by the National Key Research and Development Program [grant number 2017YFC1309200, 2022YFC2009600] and the Beijing Municipal Science and Technology Commission [grant number SCW2018-06].

## Conflict of interest

The authors declare that the research was conducted in the absence of any commercial or financial relationships that could be construed as a potential conflict of interest.

## Publisher’s note

All claims expressed in this article are solely those of the authors and do not necessarily represent those of their affiliated organizations, or those of the publisher, the editors and the reviewers. Any product that may be evaluated in this article, or claim that may be made by its manufacturer, is not guaranteed or endorsed by the publisher.
