# Supplementary material for: The combination of hand grip strength and modified Glasgow prognostic score predicts clinical outcomes in patients with liver cancer
Source: Front Nutr. 2023 Feb 27;10:1062117. doi: 10.3389/fnut.2023.1062117 (PMC10008921; doi:10.3389/fnut.2023.1062117)
Supplement: Supplementary file 1 [file Data_Sheet_1.PDF]

# *Supplementary Material*

## Supplementary Figures and Tables

### Supplementary Tables

**Table S1.** Baseline Characteristics of Patients(n=504) with Liver Cancer According to Different HGS and mGPS Levels.

| | **HGS** | | | **mGPS** | | |
|---|---|---|---|---|---|---|
| | High HGS(n=256) | Low HGS(n=248) | P-value | Low mGPS(n=148) | High mGPS(n=356) | P-value |
| Sex (%) | | | 1.00 | | | 0.79 |
| Man | 196 (76.6) | 190 (76.6) | | 115(77.7) | 271 (76.1) | |
| Woman | 60 (23.4) | 58 (23.4) | | 33 (22.3) | 85 (23.9) | |
| Age (mean (SD)) | 52.56 (11.53) | 60.83 (11.14) | <0.001 | 54.63 (10.50) | 57.46 (12.58) | 0.016 |
| Tumor Stage (%) | | | 0.001 | | | <0.001 |
| I | 45 (17.6) | 32 (12.9) | | 34 (23.0) | 43 (12.1) | |
| II | 59 (23.0) | 34 (13.7) | | 40 (27.0) | 53 (14.9) | |
| III | 55 (21.5) | 47 (19.0) | | 27 (18.2) | 75 (21.1) | |
| IV | 97 (37.9) | 135 (54.4) | | 47 (31.8) | 185 (52.0) | |
| Surgery, n (%) | | | 0.733 | | | <0.001 |
| Yes | 106 (41.4) | 98 (39.5) | | 80 (54.1) | 124 (34.8) | |
| No | 150 (58.6) | 150 (60.5) | | 68 (45.9) | 232 (65.2) | |
| Chemotherapy, n (%) | | | 0.966 | | | 0.079 |
| Yes | 43 (16.8) | 43 (17.3) | | 18 (12.2) | 68 (19.1) | |

| | | | | | | |
|---|---|---|---|---|---|---|
| No | 213 (83.2) | 205 (82.7) | | 130 (87.8) | 288 (80.9) | |
| Radiotherapy, n (%) | | | 0.080 | | | 0.046 |
| Yes | 5 (2.0) | 13 (5.2) | | 1 (0.7) | 17 (4.8) | |
| No | 251 (98.0) | 235 (94.8) | | 147 (99.3) | 339 (95.2) | |
| Smoking (%) | | | 0.004 | | | 0.030 |
| Yes | 73 (28.5) | 49 (19.8) | | 35 (23.6) | 87 (24.4) | |
| No | 40 (15.6) | 66 (26.6) | | 22 (14.9) | 84 (23.6) | |
| Other | 143 (55.9) | 133 (53.6) | | 91 (61.4) | 185 (52.0) | |
| Alcohol n (%) | | | 0.260 | | | 0.296 |
| Yes | 60 (23.4) | 70 (28.2) | | 33 (22.3) | 97 (27.2) | |
| No | 196 (76.6) | 178 (71.8) | | 115(77.7) | 259 (72.8) | |
| Albumin (g/L) | 38.48 (6.38) | 35.51 (5.49) | <0.001 | 41.27 (3.55) | 35.25 (6.13) | <0.001 |
| BMI (kg/m2) | 23.16 (3.26) | 21.55 (2.99) | <0.001 | 22.94 (2.94) | 22.13 (3.32) | 0.010 |
| Total protein (g/L) | 69.28 (7.78) | 67.42 (8.30) | 0.010 | 71.21 (5.42) | 67.18 (8.70) | <0.001 |
| CRP (mg/L) | 13.40 [3.33, 28.22] | 21.92 [6.79, 42.72] | <0.001 | 2.88 [0.99, 5.00] | 26.62 [15.30, 41.77] | <0.001 |
| AST (U/L) | 35.15 [26.00, 59.17] | 49.10 [28.85, 91.00] | <0.001 | 34.60 [26.00, 45.45] | 47.00 [28.00, 90.75] | <0.001 |
| ALT (U/L) | 34.12 [23.23, 55.25] | 38.30 [23.08, 62.12] | 0.465 | 32.00 [22.88, 41.82] | 39.00 [23.48, 69.00] | 0.002 |
| TSF (mm) | 16.00 [12.00, 21.22] | 12.00 [8.38, 17.00] | <0.001 | 15.50 [12.00, 19.31] | 14.00 [10.00, 20.00] | 0.061 |
| HGS(kg) | 31.95 [28.67, 36.00] | 21.45 [16.08, 25.30] | <0.001 | 28.00 [23.08, 33.05] | 25.00 [19.20, 31.20] | <0.001 |

**Notes:** Low HGS includes HGS<28.3 in men and HGS<18.6 in women. High HGS includes HGS≥28.3 in men and HGS≥18.6 in women. Low mGPS is mGPS=0, high mGPS includes mGPS=1 and mGPS=2.

Continuous variables are presented as mean ± standard deviation (SD). Meanwhile, TSF, HGS, CRP, AST and ALT are presented as the median (interquartile range). Categorical variables are presented as numbers and percentages. Differences in normally and non-normally distributed baseline characteristics were compared using the chi-square test or t-test and Wilcoxon rank sum test, respectively. TSF, triceps skinfold thickness; HGS, handgrip strength; BMI, body mass index; CRP, C reactive protein; AST, aspartate aminotransferase; ALT, alanine aminotransferase.

**Table S2. Association of Each Indicator and Overall Survival in Patients with Liver Cancer according to Cox Regression Models.**

| | **Univariate analysis** | | **Multivariate analysis** | |
|---|---|---|---|---|
| | HR (95%CI) | P-value | HR (95%CI) | P-value |
| **Age** | **1.02(1.01-1.03)** | **0.002** | 1.01(1.00-1.03) | **0.011** |
| **Sex** | 0.93(0.70-1.26) | 0.656 | 0.86(0.62-1.19) | 0.361 |
| **Tumor stage** | **1.76(1.54-2.01)** | **<0.001** | **1.59(1.38-1.84)** | **<0.001** |
| **Smoking** | **1.07(1.00-1.15)** | **0.040** | 0.98(0.91-1.05) | 0.540 |
| **Alcohol consumption** | 1.14(0.87-1.51) | 0.345 | 1.01(0.75-1.36) | 0.947 |
| **BMI** | **0.93(0.89-0.97)** | **<0.001** | 0.98(0.94-1.03) | 0.452 |
| **TSF** | **0.98(0.96-0.99)** | **0.007** | 0.99(0.97-1.01) | 0.351 |
| **AST** | **1.00(1.00-1.00)** | **0.001** | 1.00(1.00-1.00) | 0.096 |
| **ALT** | 1.00(1.00-1.00) | 0.686 | 1.00(1.00-1.00) | 0.721 |

**Notes:** Data are presented as hazard ratios (95% confidence intervals). Multiple models adjust for age, sex, tumor stage, smoking, alcohol consumption, BMI, TSF, unless they were independent variables themselves. HR, hazard ratio; 95%CI, 95% confidence intervals; BMI, body mass index; TSF, triceps skinfold thickness; AST, aspartate aminotransferase; ALT, alanine aminotransferase. Bold values indicate statistically significant level ($p < 0.05$).

**Supplementary Figures**

**Supplementary Figure 1.** The figure legends are required to have the same font as the main text, 12 point normal Times New Roman, single spaced. Please use a single paragraph for each legend and prepare the figures keeping in mind the PDF layout.

**Figure S1.** Flow chart.

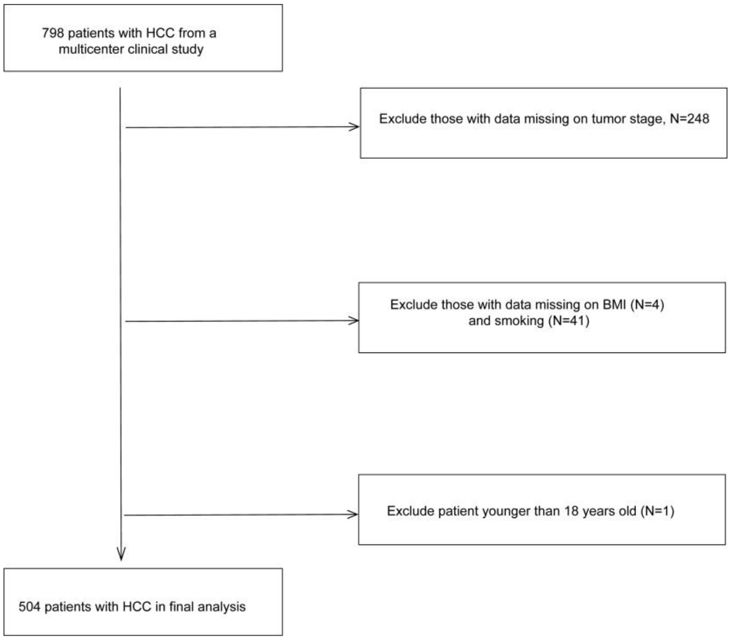

**Figure S2.** Cutoff Values of HGS.

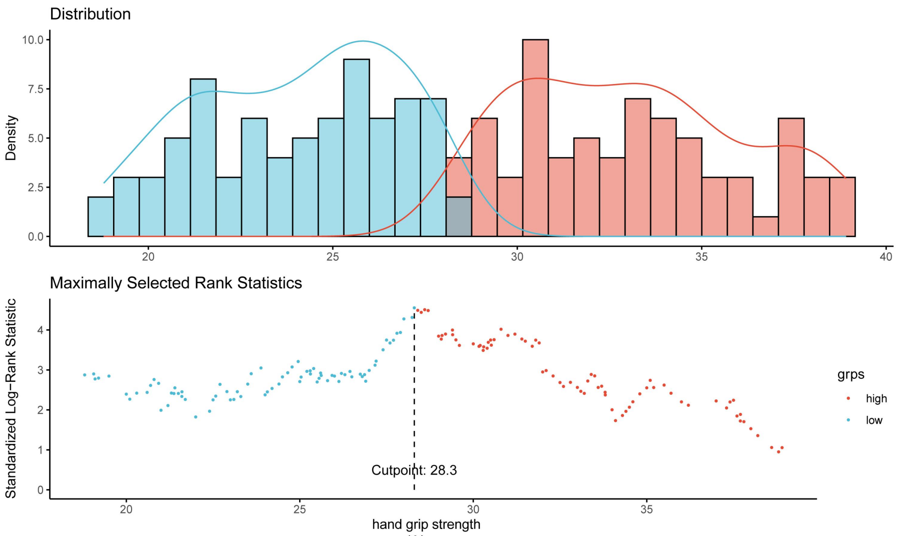


(A)

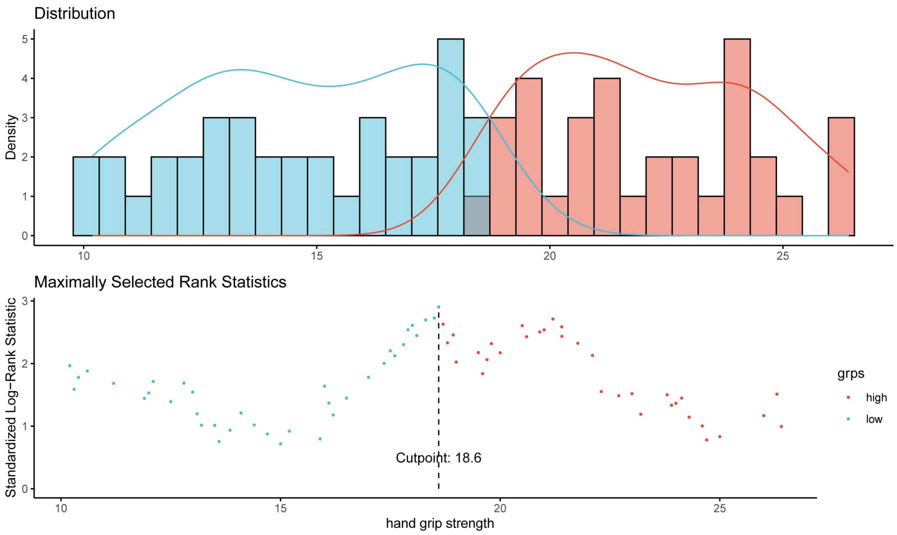


(B)

**Figure S3.** Receiver operating characteristic (ROC) curve

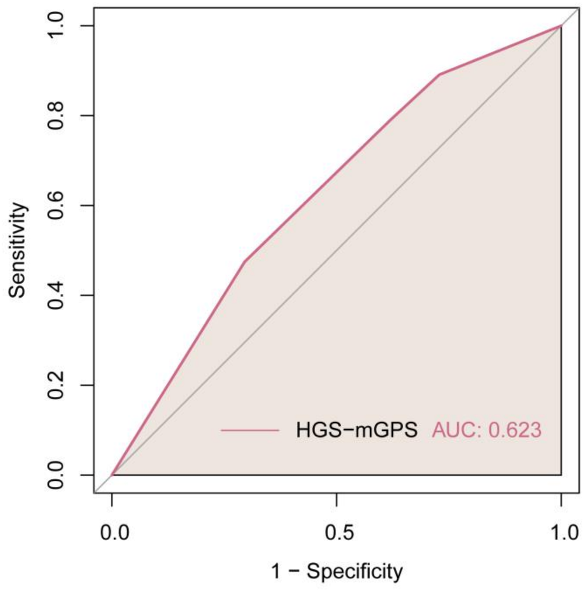


**Figure S4.** Calibration Curves for 1-year and 5-year survival

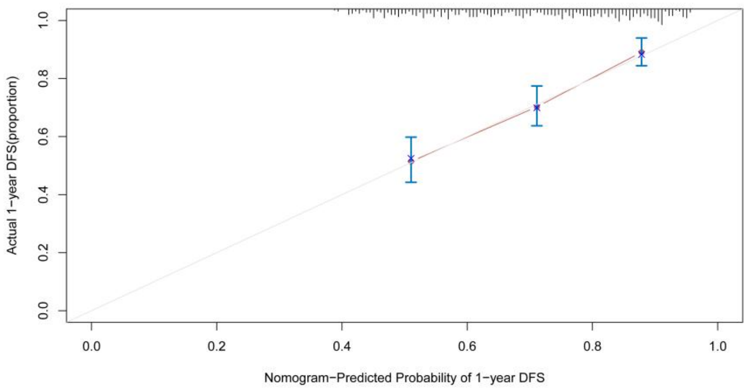


(A)

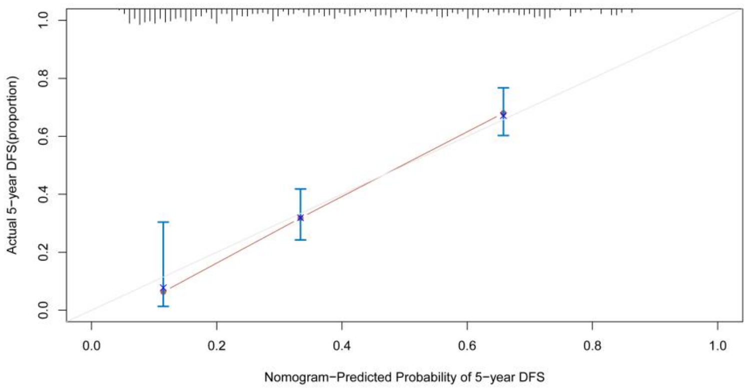


(B)
